# Association Between Sex Steroids and Oxidative Status with Vitamin D Levels in Follicular Fluid of Non-obese PCOS and Healthy Women

**Published:** 2019

**Authors:** Fatemeh Masjedi, Sara Keshtgar, Fatemeh Agah, Narges Karbalaei

**Affiliations:** - Department of Physiology, School of Medicine, Shiraz University of Medical Sciences, Shiraz, Iran

**Keywords:** Oxidative stress, Polycystic ovary syndrome, Sex steroid hormones, Vitamin D

## Abstract

**Background:**

Human follicular fluid (FF) is rich in hormones and antioxidants. Many components of FF differ in follicles of patients with polycystic ovary syndrome (PCOS). Regarding vitamin D effects on gene expression, 25(OH)D level of FF and its association with oxidative status and sex steroids dysregulation in PCOS group was evaluated and compared to controls of Non-obese healthy women.

**Methods:**

FF of 50 non-obese healthy women and 50 women with PCOS (18–36 years old) who were candidates for IVF/ICSI was aspirated on the oocyte retrieval day. Sex steroids and 25(OH)D levels were measured by ELISA. Reactive oxygen species (ROS) levels, total antioxidant capacity (TAC), and activities of superoxide dismutase (SOD), glutathione peroxidase (GPx), and catalase (CAT) were assessed by chemiluminescence and spectrophotometric methods. Data were analyzed by unpaired t-test or Mann-Whitney test, and Pearson correlation coefficient. The p<0.05 was considered statistically significant.

**Results:**

Estradiol, progesterone, 25(OH)D, TAC, and activities of SOD, GPx, and CAT in FF of women with PCOS were significantly lower, whilst their free and total testosterone and ROS levels were significantly higher than controls. There were significant positive correlations between FF levels of 25(OH)D with TAC, estradiol and progesterone concentrations, SOD, GPx, and CAT activities. Negative correlations were found between 25(OH)D with free and total testosterone, and ROS levels.

**Conclusion:**

Despite different hormonal and antioxidant levels in FF of normal and cystic follicles, the correlation between 25(OH)D levels with sex steroids and oxidative stress markers showed a possible role of 25(OH)D in regulating sex hormones secretion and enhancement of antioxidant defense.

## Introduction

Polycystic ovary syndrome (PCOS) is one of the most common hormonal disorders that affects women during reproductive period. This syndrome is characterized by hyperandrogenism, polycystic ovarian morphology, and ovulatory impairment (1). High percentages (55–75%) of women with PCOS have an elevated luteinizing hormone (LH) to follicle-stimulating hormone (FSH) ratio, which presumably is due to hypersecretion of LH rather than diminished production of FSH. Normally, LH stimulates androgen synthesis in the theca cells, and aromatase enzyme converts androgens into estrogens. However, an androgen rich environment favors the conversion of testosterone to another potent androgen (dihydrotestosterone) instead of estrogens. In fact, excess amount of androgens inhibit aromatase activity, leading to deficiency in estrogen production. In addition, there are some abnormalities in progesterone (P4) synthesis in patients with PCOS that might be related to high rate of abortion (2).

Vitamin D is a lipid soluble steroid molecule and its receptor (VDR) has been identified in the female reproductive tissues including human ovaries, endometrium, fallopian tube, placenta, and decidual cells (3). Several studies have suggested that vitamin D deficiency is more common among women with PCOS compared to healthy subjects (4–6). There is evidence proposing that vitamin D deficiency might contribute in the pathogenesis of insulin resistance and the metabolic dysfunction in PCOS (7, 8). Some correlations between low serum 25(OH)D status with features of PCOS have been shown. Vitamin D deficiency was associated with an imbalance in serum dehydroepiandrosterone (DHEA), sex hormone binding globulin (SHBG), testosterone (T), and free androgen index (FAI) (9–11). In a study on women with PCOS, a significant correlation was found between 25(OH)D levels and LH/FSH ratio (12). Furthermore, vitamin D regulates estradiol (E2) biosynthesis by directly affecting the aromatase gene. Its deficiency leads to reduced aromatase activity in the ovary (13, 14).

In addition to endocrine disturbances, PCOS is also associated with oxidative stress (OS) that impairs female fertility function (15, 16). Abnormal circulating OS markers were observed in women with PCOS independent of excess weight. It has been suggested that OS might contribute to some aspects of this syndrome pathophysiology (17). Several animal studies have indicated that vitamin D might have antioxidant properties by modifying some antioxidant defense enzymes (18, 19). There are limited data about the association between 25(OH)D levels with OS markers (20, 21). Recently, a study investigated the associations between serum 25(OH)D levels with malondialdehyde (MDA) and protein carbonyl (PC) in women with PCOS, but no correlation was found between the serum vitamin D levels and the mentioned OS markers (22).

Actually, most researches have focused on serum levels of vitamin D and other hormones in both women with PCOS and healthy ones. As far as we know, there is no study on the correlation of vitamin D in follicular fluid (FF) with steroid hormone synthesis or antioxidant defense properties of human follicular cells. Studying FF as an important strategic fluid is imperative, because the human FF forms a metabolically active microenvironment for developing the oocyte and granulosa cells, which has an important influence on embryonic development (23). Human FF originates from the diffusion of blood components through thecal capillaries and by granulosa, theca cells and oocyte secretion products are obtained (24). Indeed, the molecular characterization of FF and functional correlation between the identified metabolites and proteins could lead to the discovery of biomarkers with diagnostic and predictive values for a wide range of fertility problems.

Previous studies focused on the relationship between vitamin D levels of FF and *in vitro* fertilization (IVF) outcomes in women with healthy or polycystic ovaries (25, 26). However, the association between 25(OH)D levels of FF with sex steroid hormones and OS markers has not been investigated in normal and PCOS women.

Therefore, the present research aimed to compare the concentration of 25(OH)D, sex steroid hormones, and OS markers in FF of normal women and patients with PCOS. In addition, the correlations between FF levels of 25(OH)D with sex steroid hormones and OS markers were evaluated, to clarify the importance of vitamin D on the follicular sex steroid biosynthesis and oxidative stress in normal women relative to women with PCOS.

## Methods

### Study subjects and follicular fluid collection:

The present study was carried out on FF of 100 women (18–36 years old) who referred to Shiraz Fertility Center between January 2017 and October 2017, for IVF or intracytoplasmic sperm injection (ICSI). Fifty women with PCOS were considered as PCOS group, and the control group consisted of 50 women with healthy ovaries diagnosed as male factor, tubal disease or unexplained infertility. The PCOS syndrome was diagnosed by a gynecologist according to Rotterdam criteria (27), which include at least two of the three criteria:

1) oligo- and/or anovulation, 2) clinical and/or biochemical signs of hyperandrogenism, and 3) polycystic ovaries (presence of 12 or more follicles in each ovary measuring 9±2 *mm* in diameter, and/or increased ovarian volume).

All participants had a body mass index (BMI) between 18.5–30 *kg/m*^2^ . Exclusion criteria included having major medical disorders (such as other hyperandrogenism states, thyroid diseases, and diabetes), taking certain medications, smoking, and alcohol consumption.

All women were prepared for IVF or ICSI, under a gynecologist’s supervision, and the gonadotropin-releasing hormone (GnRH) antagonist protocol was the main treatment option used in both groups (28). Briefly, controlled ovarian hyperstimulation was initiated by daily injection of 150–300 *IU* recombinant human FSH (Gonal-F®, Merck-Serono, Germany) from the second/third day of their menstrual cycle. When at least one follicle reached 12–14 *mm* in diameter, the GnRH antagonist (Cetrotide®, Merck-Serono) was administered once a day, until at least three follicles of ≥17 *mm* were observed. At this time, recombinant human chorionic gonadotropin (hCG) (Ovitrelle®, Merck-Serono) was administered to induce final oocyte maturation. Transvaginal-guided follicular puncture was performed and FF was aspirated under general anesthesia 34–36 *hr* after administration of hCG into a test tubes. An embryologist isolated and removed the oocytes from the fluid and the remaining follicular aspirates from each patient were pooled and transported to the laboratory in less than an hour at temperature 4°*C*.

The local Ethics Committee of Shiraz University of Medical Sciences, Shiraz, Iran approved this study (EC. 9370. 7320). All the experiments involving human participants were performed in accordance with the 1964 Helsinki declaration and relevant guidelines. Written informed consents were obtained from all women who participated in our research study.

### Follicular fluid preparation:

Follicular aspirates were transferred to 50 *ml* sterile conical centrifuge tube and centrifuged at 1600 *rpm* for 10 *min* in room temperature (22–25°*C*). The supernatant fluid as FF was then collected, reactive oxygen species (ROS) level detected and the reminder stored at −20°*C* for further analysis.

### ROS measurement:

Amount of ROS production was measured by luminol/horseradish peroxidase (HRP)-dependent chemiluminescence assay. To determine the ROS levels, 300 *μl* fresh FF sample was transferred to a black 96-well plate. Luminol with concentration of 25 *mM* and 12 *IU/ml* HRP were added and the Relative Light Unit (RLU) was assessed for 30 *min* with 2-*min* intervals at 37°*C* using a microplate reader with luminescence detection technology (Synergy TM HT, BioTek® instruments, Inc. Winooski, Vermont, USA). The blank well contained PBS, luminal and HRP. The RLU of blank well was subtracted from sample wells. Experiments were performed in duplicate.

### 25(OH)D and sex steroid hormones assay in follicular fluid:

Levels of 25(OH)D, E2, P4, free testosterone (free T), and total testosterone (total T) were measured in FF of normal women and patients with PCOS. These assays were carried out by commercial ELISA kits [Diagnostics Biochem Canada Inc. for sex hormones and Orgentec Company for 25(OH)D] according to the manufacturer’s recommendations using a microplate reader (ELx808TM, BioTek® instruments, USA).

### TAC assay:

Biological fluids have an array of protective antioxidant mechanisms, both for preventing the production of free radicals and for repairing oxidative damage. In the present study, TAC assay was performed based on thiobarbituric acid reactive substances (TBARS) method (29).

Briefly, a test reaction mixture containing 490 *μl* of PBS (100 *mmol/L*), 500 *μl* of Na- benzoate (10 *mmol/L*), 200 *μl* of Fe–EDTA complex (2 *mmol/L*), 200 *μl* of hydrogen peroxide (H_2_O_2_) (10 *mmol/L*) with 10 *μl* of FF sample was incubated for 60 *min* at 37*°C*. Then, 1 *ml* of 20% acetic acid and 1 *ml* of thiobarbituric acid (TBA) (0.8% (wt./vol.) in 50 *mmol/L* NaOH) were added to the test tube. After 10 *min* of incubation at 100°*C* (in a boiling water bath) and cooling (in an ice bath), absorbance was measured at 532 *nm* against deionized water using a microplate reader. Each sample (A1) had its own control (A0) in which the Fe–EDTA mixture and H_2_O_2_ should be added after 20% acetic acid. For each series of analysis, a negative control was prepared (at least in duplicate), containing the same reagents as A1 or A0, except that FF sample was replaced with PBS. A solution of 1 *mmol/L* uric acid was used as standard.

Based on TBARS test principle, a standardized solution of Fe–EDTA complex reacts with H _2_ O _2_ by a Fenton-type reaction, leading to the formation of hydroxyl radicals. These radicals degrade benzoate, resulting in the release of TBARS. Antioxidants from the added FF sample cause suppression of the production of TBARS. The inhibition of color development was defined as the TAC.

### Antioxidant enzymes activity assay:

(a) The superoxide dismutase (SOD) activity in FF was measured according to the Misra and Fridovich procedure (30). This method is based on the inhibition of the auto-oxidation of epinephrine to adrenochrome by SOD at pH=10.25. Briefly, reaction mixtures containing 925 *μl* 0.05 *M* bicarbonate buffer (Na2 CO3/NaHCO3) (pH=10.25) with 10 ^−4^ *M* EDTA and 50 *μl* of FF sample were added to the cuvette. One minute after the addition of 25 *μl* of 0.01 *M* epinephrine in 0.02 *M* HCl, light absorbance by adrenochrome was read at 485 *nm*, every 30 *s* for duration of 10 *min* using spectrophotometer (Spectrolab UV 7500, Newbury, Berkshire, England). Serial dilution from standard SOD was prepared in K ^+^ -phosphate buffer (0.1 *M*, pH=7.4). The percentage inhibition of epinephrine auto-oxidation was plotted against various concentrations of standard SOD. The amount of enzyme, which produces 50% inhibition of epinephrine auto-oxidation, was considered as one unit of the enzyme activity. The SOD activity was reported as *unit/ml* of FF.

(b) The glutathione peroxidase (GPx) activity in FF was measured according to the modified method of Fecondo and Augusteyn (31). Briefly, 142 *μl* of reaction mixture containing 0.3 *mmol/L* ethylenediaminetetraacetic acid (EDTA), 0.1 *mmol/L* β-nicotinamide adenine dinucleotide 2′-phosphate (β-NADPH), 0.5 units of glutathione reductase (GR) and 0.5 *mmol/L* sodium azide (Na2N3) in 50 *mmol/L* PBS (pH=7.0), 20 *μl* of FF sample, and 10 *μl* of 2.5 *mmol/L* L-Glutathione reduced (GSH) were added to each well of a 96-well plates. Wells in which distilled water was substituted for GSH were considered as the controls. Following the addition of 20 *μl* of 0.4 *mmol/L* tert-Butyl hydroperoxide (t-BuOOH), the decrease in NADPH absorbance at 340 *nm* was measured at 25°*C*, every minute for duration of 10 *min* using a microplate reader. The rate of ΔA _340_ /*min* was determined for GSH-negative wells (control) and this rate was subtracted from the corresponding GSH-containing wells. The NADPH extinction coefficient was adjusted for the path length (0.6 *cm*) of the solution in the well to 0.00377 *μ*M ^−1^ according to 0.00622 *μ*M ^−1^*cm*^−1^ for usual spectrophotometer.

One unit of GPx activity is defined as the amount of enzyme that will cause oxidation of 1.0 *μ*mol of NADPH to NADP ^+^ per minute at 25°*C*, pH=7.0. The GPx activity in FF was expressed as *μ*mol of NADPH *oxidized/min/ml* of FF.

(c) The catalase (CAT) activity was assayed by measuring the rate of disappearance of H _2_ O _2_ spectrophotometrically (32). To begin the assay, 1 *ml* of the H _2_ O _2_ solution (10 *mM*) was added to a reaction mixture containing 1.9 *ml* of PBS and 100 *μl* of FF sample. After the content mixing, quartz cuvette was immediately placed into the spectrophotometer (Genesys 5, Thermo Spectronic, USA). The decrease in absorbance of H _2_ O _2_ was recorded at 240 *nm* every 15 *s* for 2 *min*. Cuvette containing 1.9 *ml* PBS and 100 *μl* of FF sample was considered as a blank per each experiment.

The CAT activity was calculated using the rate of ΔA _240_/*min*. and molar extinction coefficient for H _2_ O _2_ (0.0436 *μ*M ^−1^*cm*^−1^). Each unit of CAT decomposes 1.0 *μmol* of H _2_ O _2_ per *min* at 25°*C* and pH=7.0. The CAT activity in FF was expressed as *μ*mol of H _2_ O _2_ /*min/ml* of FF.

### Statistical analysis:

Data were checked for normality using Shapiro–Wilk test. Comparisons were performed using unpaired t-test if the data were normally distributed and if the data were not normally distributed, Mann-Whitney test was used. The correlations between continuous quantitative and qualitative variables were shown by the Pearson correlation coefficient. Using Fisher r-to-z transformation, Z value was calculated that can be applied to assess the significance of the difference between two correlation coefficients, r _a_ and r _b_ , found in two independent samples. All the categorical variables were compared with a Pearson Chi-square test between the two groups. Demographic and experimental data were expressed as mean±SEM. The level of statistical significance was set at p<0.05 for all statistical analyses. Data were analyzed in GraphPad Prism for Windows (version 6.0, GraphPad Software Inc. La Jolla, California, USA).

## Results

Basal characteristics of control and PCOS groups are shown in table 1. Statistical analysis showed no significant differences in age, BMI, and duration of infertility between the two groups. The frequency of menstrual irregularities in PCOS was significantly (p<0.0001) greater than controls.

**Table 1.** Demographic characteristic of participants in the control and PCOS groups

**Parameters**	**Control group (n=50)**	**PCOS Group (n=50)**	**p-value**
**Age (years)**	29.85±0.514	29.47±0.465	0.530 ^*^
**BMI (*kg/m*^2^)**	24.97±0.473	25.22±0.442	0.927 ^*^
**Duration of infertility (years)**	4.36±0.498	5.16±0.527	0.354 ^*^
**Menstrual status**			
Regular	42 (84%)	17 (34%)	0.0001 ^**^
Irregular	8 (16%)	33 (66%)	0.0001 ^**^

* Unpaired t-test,

** Pearson Chi-square test. Data are presented as mean±SEM and n (%). BMI: Body Mass Index

### Sex steroid hormones and 25(OH)D levels:

The FF levels of sex steroid hormones and 25(OH)D in women with PCOS and normal subjects are shown in table 2. The mean levels of E2 and P4 in FF of women with PCOS were significantly (p<0.0001) lower and free and total T were significantly (p< 0.0001) higher than the controls. The mean FF level of 25(OH)D was significantly lower in patients group in comparison with control group.

**Table 2.** Follicular fluid levels of 25(OH)D and sex steroid hormones in the control and PCOS groups

**Parameters**	**Control group (n=44)**	**PCOS Group (n=44)**	**p-value**
**Estradiol (*ng/ml*)**	1485.80±76.09	466.10±26.73	0.0001 ^*^
**Progesterone (*ng/ml*)**	10487.71±44.73	7660.98±125.00	0.0001 ^*^
**Free testosterone (*pg/ml*)**	21.79±0.92	50.73±0.70	0.0001 ^**^
**Total testosterone (*ng/ml*)**	6.61±0.08	14.31±0.30	0.0001 ^**^
**25(OH)D (*ng/ml*)**	41.77±2.81	23.02±2.03	0.0001 ^*^

* Mann-Whitney test,

** Unpaired t-test. Data are presented as mean±SEM

### ROS levels, TAC, and activities of SOD, GPx, and CAT:

The FF levels of ROS, TAC, and antioxidant enzymes activity in normal women and patients with PCOS are shown in table 3. The amount of ROS in FF of women with PCOS was significantly (p<0.0001) higher than normal. The average FF TAC and activities of SOD, GPx, and CAT enzymes in patients group were significantly (p<0.0001) lower than the control group.

**Table 3.** Follicular fluid ROS levels, TAC, and activities of SOD, GPx, and CAT in the control and PCOS groups

**Parameters**	**Control group (n=50)**	**PCOS Group (n=50)**	**p-value**
**ROS (RLU)**	117.14±2.96	514.12±19.83	0.0001 ^*^
**TAC (*mmol/l***)	1.40±0.01	1.15±0.03	0.0001 ^*^
**SOD activity (*IU/ml*)**	1.76±0.02	1.60±0.02	0.0001 ^**^
**GPx activity (*IU/ml*)**	56.27±3.64	37.48±1.64	0.0001 ^*^
**CAT (*IU/ml*)**	8.94±0.23	5.53±0.14	0.0001 ^*^

* Mann-Whitney test,

** Unpaired t-test. Data are presented as mean±SEM. ROS: Reactive Oxygen Species, RLU: Relative Light Unit, TAC: Total Antioxidant Capacity, SOD: Superoxide Dismutase, GPx: Glutathione Peroxidase, CAT: Catalase

### Correlation between FF levels of 25(OH)D with demographic variables:

In the case of correlation assessment between variables, no significant correlation was observed between 25(OH)D levels in FF with age and BMI, whereas statistically significant negative correlation was found between 25(OH)D levels with duration of infertility in the studied groups. In addition, women with menstrual irregularities exhibited significantly lower FF 25(OH)D level in the control and PCOS groups (Table 4).

**Table 4.** Correlation between FF levels of 25(OH)D with demographic characteristics in the control and PCOS groups

**Variables**	**Control Group (n=50)**		**PCOS group (n=50)**	

	**R**	**p-value**	**r**	**p-value**
**Age (years)**	−0.124	0.427	−0.166	0.277
**BMI (*kg/m*^2^)**	−0.178	0.252	−0.164	0.290
**Duration of infertility (years)**	−0.415	0.005	−0.357	0.016
**Menstrual irregularity (Oligomenorrhea)**	−0.639	0.0001	−0.768	0.0001

r= Pearson correlation coefficient, BMI: Body Mass Index

### Correlation between FF levels of 25(OH)D with sex steroid hormones:

**Figure 1.**
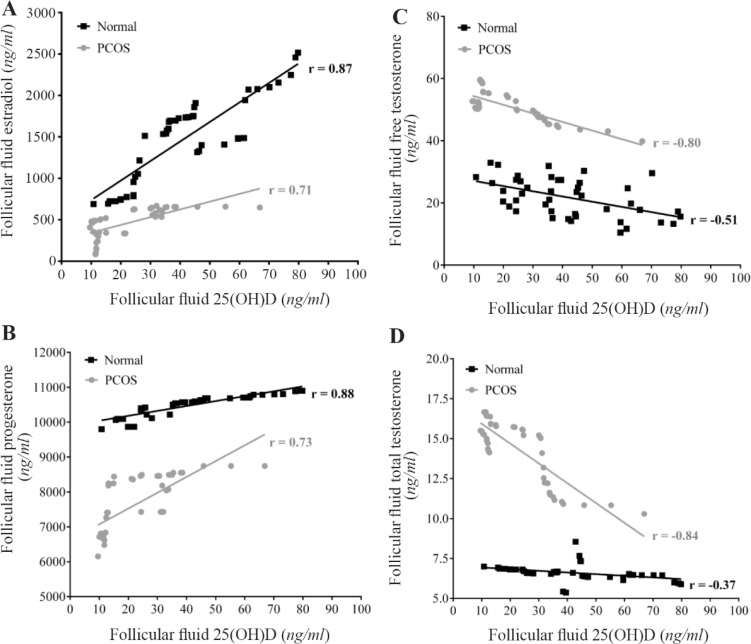
Correlation between follicular fluid levels of 25(OH)D with Estradiol (A), Progesterone (B), free Testosterone (C), and total Testosterone (D) in the control (n=44) and PCOS (n=44) groups. There were significant positive correlations between FF levels of 25(OH)D with E2 and P4, while significant negative correlations were observed between FF levels of 25(OH)D with free and total testosterone in the two studied groups. r= Pearson correlation coefficient (p<0.001)

Figure 1 presents the correlation between FF levels of 25(OH)D with sex steroid hormones in the two studied groups. In the control and PCOS groups, significant positive correlations were found between FF levels of 25(OH)D with E2 and P4, while significant negative correlations were observed between FF levels of 25 (OH)D with free T and total T. Calculated Z values for E2, P4, free T, and total T were 2.02, 2.02, 2.43, and 3.77, respectively. Z value ≥2 represents significant difference between the two correlation coefficients (r) in control and PCOS groups.

### Correlation between FF levels of 25(OH)D with ROS, TAC, and antioxidant enzymes activities:

**Figure 2.**
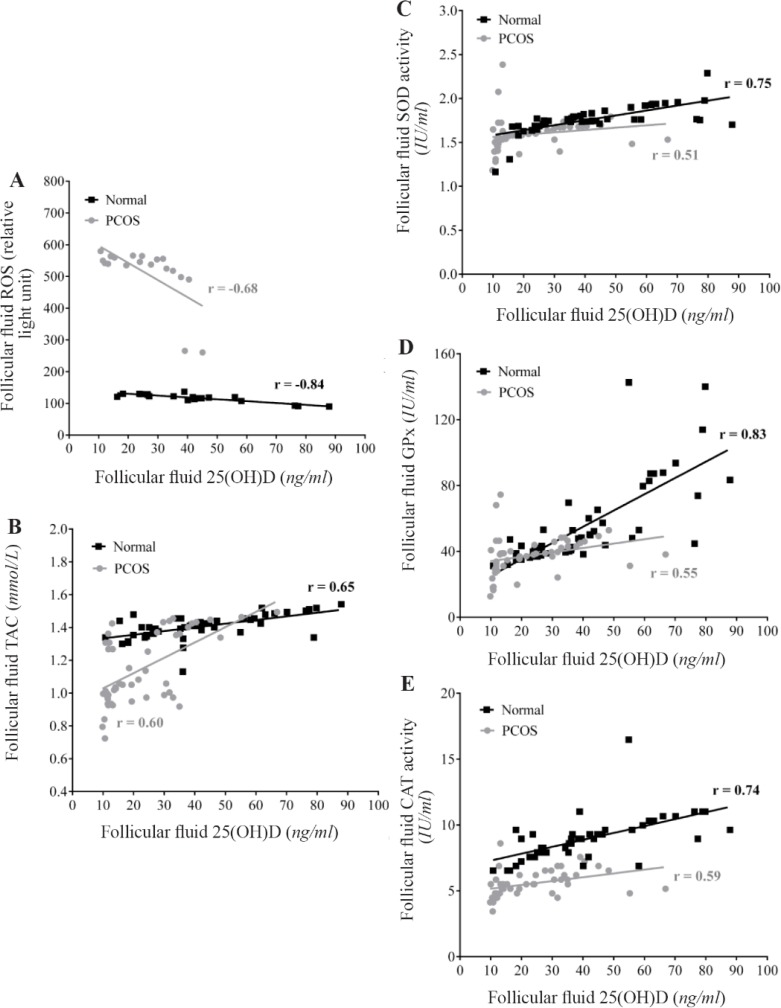
Correlation between follicular fluid levels of 25(OH)D with ROS (A), TAC (B), SOD (C), GPx (D) and CAT (E) in the control (n=50) and PCOS (n=50) groups. There were significant positive correlations between FF levels of 25(OH)D with TAC and activities of SOD, GPx, and CAT, while a significant negative correlation was found between FF levels of 25(OH)D with ROS amounts in the two studied groups. r= Pearson correlation coefficient (p<0.001)

Figure 2 shows the correlation between FF levels of 25(OH)D with ROS, TAC, and antioxidant enzymes activity (SOD, GPx, & CAT) in both studied groups. 25(OH)D in FF of normal women and patients with PCOS positively correlated with TAC and activities of SOD, GPx and CAT, whereas, a significant negative correlation was found between FF 25(OH)D and ROS levels. Calculated Z values for ROS, TAC, SOD, GPx, and CAT were −1.14, 0.4, 2, 2.76, and 1.32, respectively. Consequently, Z values showed significant differences between two correlation coefficients (r) for SOD and GPx activities in control and PCOS groups.

## Discussion

In this study, 84% of normal women had regular menstrual cycle, while only 34% of women with PCOS experienced regular cycle and more than 65% of them had oligomenorrhea. Another study on 1741 women showed the normal regular menses in 30% of women with PCOS (33). It was shown that almost 85%–90% of women with oligomernorrhea have PCOS (34). Among the PCOS population, obese women revealed lower 25(OH)D levels compared to overweight or lean subjects (35). Our study was performed on non-obese women, and the data showed a negative correlation between FF 25(OH)D and BMI of the participants, although statistically not significant. Vitamin D supplementation was shown to improve menstrual cycle and metabolic disturbances in women with PCOS (36).

Over the past decade, the physiological role of 25(OH)D has been evaluated extensively (37), and despite its role in human reproduction, the data in this issue are scarce. VDRs have been identified in endometrium, and Ozkan et al. (2010) reported that replete FF vitamin D levels predict the IVF outcome, recommending the beneficial role of sufficient or additional vitamin D concentrations in endometrial receptiveness (25). These findings are in agreement with the present study regarding the association of vitamin D with the menstrual regularity and its possible beneficial effects on endometrium and the ovarian hormonal balance.

The sex steroids are synthesized by follicular cells during the follicle maturation procedure and they accumulate in FF. Since, the corona-cumulus- oocyte complex is in close contact with FF, an association is assumed to exist between the hormonal content of FF and the quality of oocyte maturity. Therefore, FF hormonal content is likely to be associated with fertilization, implantation rate and embryo development (38, 39).

In the present study, women with PCOS showed a significant reduction in 25(OH)D, E2 and P4 level in FF, when compared with normal women. The follicular levels of free T and total T were significantly higher in women with PCOS. In agreement with our results, other studies showed that in women with PCOS, ovarian FF contained higher concentrations of free and total androgens, lower estrogens, and a lower estrogen-to-androgen ratio, compared with regularly menstruating women (40, 41). The results of another study showed the follicular estrogen remained constant in PCOS patients and follicular P4 levels were markedly reduced (42).

Studies on the association of vitamin D with hormones in the human FF are sparse. One study showed a positive non-significant correlation between FF vitamin D levels and serum E2 levels (43). Our results showed that women with higher levels of FF 25(OH)D had higher levels of E2 and P4, while their T levels decreased in comparison with those who had low FF 25(OH)D levels. Vitamin D acts as a transcription factor regulating the expression of CYP19 gene. This gene encodes aromatase, which is the main enzyme in the androgens to estrogens conversion (13). It is concluded that vitamin D may attenuate the androgenic milieu of cystic follicles and reduce the complications of PCOS.

Other observational studies investigated the association of serum 25(OH)D status with metabolic and endocrine parameters of PCOS (7, 8, 11, 44, 45). In general, women with PCOS had lower serum 25(OH)D levels compared to healthy controls. In one study on 120 women with PCOS, serum 25(OH)D levels were significantly correlated with FAI and SHBG, but not with testosterone, DHEAS, androstenedione and LH/FSH ratio (9). In the subsequent studies, 25(OH)D levels were positively associated with SHBG and negatively associated with FAI (4, 8). On the other hand, a study on 100 women with PCOS showed that serum 25(OH)D levels were negatively correlated with testosterone and DHEAS levels in obese patients with PCOS (11). Vitamin D might regulate serum androgen levels of patients with PCOS. It has been suggested that SHBG decreases with vitamin D deficiency, and free androgen levels increase and facilitate the basis for PCOS (46). However, a recent study found no association between 25(OH)D levels and hyperandrogenism markers (47).

Nonetheless, it has been reported that addition of vitamin D supplements for treatments of patients with PCOS, improved their hormonal dysregulations, folliculogenesis defects, and menstrual irregularities (36). These results confirm the plausible role of this vitamin in female reproduction. Our finding showed the importance of vitamin D in FF in regulation of sex steroid biosynthesis as well.

On the other hand, the present study showed a significant increment in ROS levels and a reduction in TAC and activities of SOD, GPx, CAT in FF of patients with PCOS. Parameters of oxidative status might be considered as metabolic activity markers within the follicle. However, higher TAC and lower ROS levels in FF are associated with higher pregnancy rate after ICSI (48). ROS are generated in the growing follicle due to high metabolic activity (49), but under normal condition, antioxidants balance the oxidative status and prevent the OS. In pathological states, disturbance in the antioxidant defense causes OS.

However, the experimental results on the anti-oxidant enzyme activities in PCOS patients are contradictory. Pekel et al. (2015) showed SOD and TAC was impaired in serum and FF of infertile women with PCOS. There was a statistically significant decrease in FF SOD activity in PCOS group compared to healthy women. The mean relative level of Cu-Zn SOD mRNA was also significantly lower in cells isolated from the FF in PCOS than the control group (50). Appasamy et al. (2008) found that there were no differences in FF TAC levels in patients with PCOS and non-PCOS women (51). Yilmaz et al. (2016) could not show a significant difference between FF TAC levels in patients with PCOS compared to normal ones (52). Alternatively, Oyawoye et al. (2009) showed an increased TAC levels in patients with PCOS (53) and a positive correlation between TAC in FF and clinical pregnancy rate in the whole group.

A few studies have indicated that there were some relationships between serum vitamin D levels and some OS markers in women under IVF treatment (54). Recent studies suggest that OS might play a major role in the pathophysiology of PCOS, but the association of vitamin D with OS is still unknown in PCOS.

Micronutrient treatment, starting from three months before the IVF cycles, protects the follicular microenvironment from OS, consequently increasing the good quality oocytes recovered at the pickup (55). Increased expression of genes responsible for enzymes involved in cellular antioxidant defense systems in chronically vitamin D deficient female mice suggests higher levels of intraovarian OS (56).

A significant positive correlation was found between 25(OH)D with TAC and activities of SOD, GPx, and CAT in FF of women with healthy and polycystic ovaries, whereas, a significant negative correlation existed between 25(OH)D and ROS level in FF of both studied groups. Even though FF 25(OH)D was significantly lower in PCOS women than the controls, this level still correlated with ROS, TAC, SOD, GPx, and CAT. Regardless of the presence or absence of this syndrome, vitamin D deficiency can exacerbate and worsen OS. The results of this study emphasize on the effect of vitamin D on TAC and activities of SOD, GPx, and CAT in FF, and its effect on counterbalancing the produced ROS.

## Conclusion

The evaluation of sex steroid hormones and oxidative status confirmed hormonal disorders and OS in women with PCOS compared to healthy controls. There was also a significant reduction in FF 25(OH)D levels in these patients compared with the healthy women. Assessing the relationships between vitamin D levels with various sex steroid hormones and OS markers in FF of healthy women or polycystic ovaries emphasizes the role of this vitamin in regulating steroid hormone levels and enhancement of antioxidant defense. Although the causes of the PCOS are complex and even unknown, but it is suggested that sufficient level of vitamin D is associated with better reproductive function and oxidative status balance.
